# Self‐regulatory control processes in youths: A temporal network analysis approach

**DOI:** 10.1002/jcv2.12200

**Published:** 2023-09-30

**Authors:** Fiorella Turri, Andrew Jones, Lauriane Constanty, Setareh Ranjbar, Konstantin Drexl, Giorgia Miano, Caroline Lepage, Kerstin Jessica Plessen, Sébastien Urben

**Affiliations:** ^1^ Division of Child and Adolescent Psychiatry Department of Psychiatry Lausanne University Hospital (CHUV) Lausanne Switzerland; ^2^ School of Psychology Liverpool John Moores University Liverpool UK; ^3^ Center of Psychiatric Epidemiology and Psychopathology Department of Psychiatry Lausanne University Hospital University of Lausanne Prilly Switzerland; ^4^ Faculty of Biology and Medicine University of Lausanne Lausanne Switzerland

**Keywords:** adolescents, ecological momentary assessment, network analyses, response inhibition, self‐regulation

## Abstract

**Objective:**

This study aimed to better understand the temporal interrelationships among self‐control, response inhibition, and anger (i.e., momentary state and rumination) on both the within‐ and between‐person levels in male adolescents.

**Method:**

We applied temporal network analyses among 62 male adolescents with a wide range of behavioral difficulties. Self‐control, momentary anger, and anger rumination were mapped by self‐report measures, whereas we measured response inhibition through an ambulatory Go/No‐go task (two measures a day—morning and afternoon—over a 9‐day period).

**Results:**

Temporal network analysis, at the within‐person level, revealed that morning measures of response inhibition, anger rumination, and self‐control were related to the corresponding measure in the afternoon. More efficient response inhibition in the morning was associated with higher self‐control in the afternoon. Higher anger rumination in the morning led to higher momentary anger in the afternoon. In a concurrent within‐person network, higher momentary anger was reciprocally associated with lower self‐control. At the between‐person level, higher momentary anger was correlated to higher anger rumination, lower response inhibition, and lower self‐control.

**Discussion:**

This study provides insight into the dynamic interactions among self‐control, response inhibition, and anger (momentary state and rumination) in male adolescents, advancing the understanding of self‐regulatory control functioning.

## Graphical abstract

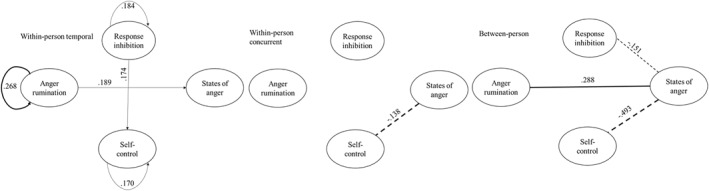

**Key points**

- We assessed the temporal dynamics of self‐control, response inhibition, and anger (momentary states and rumination) at both the within‐ and between‐person levels.
- Temporal network analyses assessed the dynamic associations among the processes measured through ambulatory assessment.
- Anger rumination precedes states of anger, whereas increased response inhibition predicted higher self‐reported self‐control over time.
- The study highlighted important temporal interrelationships at within‐ and between‐person levels among self‐control, response inhibition and experiences of anger that can inform the development of ecological momentary or just‐in‐time adaptive interventions.

## INTRODUCTION

The vast majority (75%) of psychiatric illnesses manifest before the age of 18 years (Kim‐Cohen et al., 2003). Out of the 12%–20% of children and adolescents impacted by psychiatric disorders, 40% of them will still need care during adulthood (Fombonne, 2005; Hofstra et al., 2002). Adolescence is thus a pivotal period that presents a unique window of opportunity to alleviate future difficulties (Fuhrmann et al., 2015).

Within this perspective, one core mechanism of human development is self‐regulatory control processes (e.g., Blair, 2002), which refer to intrinsic processes that allow an individual to adapt emotion, cognition, and behavior to changing environments and/or to achieve long‐term goals (Nigg, 2017). Self‐regulatory control processes are remarkable predictors of positive outcomes, such as academic achievement (Pandey et al., 2018), health (Smithers et al., 2018), and harmonious interpersonal relationships (Robson et al., 2020). By contrast, deficits in self‐regulatory control processes have been consistently related to behavioral problems (Heatherton & Wagner, 2011). Behavioral problems (e.g., aggressive and delinquent behaviors) are more prevalent in male individuals (Erskine et al., 2013; Maughan et al., 2004; Seedat et al., 2009). Moreover, girls not only display less problematic behaviors but also showed different trajectories (e.g., early onset/life course persistent, childhood limited, adolescence limited, adulthood onset) (Odgers et al., 2008) that may mirror different psychological processes (e.g., lower impulsivity traits) in girls compared with boys (e.g., Meier et al., 2008).

Self‐regulatory control processes represent an umbrella term embracing processes and components used interchangeably in the literature (e.g., executive functions, emotional regulation, and effortful control, self‐control, inhibitory control, and cognitive control). In this study, we focus on three specific components of self‐regulatory control processes—namely, self‐control, response inhibition, and rumination (i.e., one non‐adaptive emotion regulation strategy)—as well as states of anger. Self‐control is widely used in the developmental literature and involves the ability to resist temptation (Diamond, 2013), including a motivational component. Response inhibition refers to an executive function sustaining the capacity to flexibly adjust cognition and behavior in a dynamic context by stopping inappropriate responses (Carter & Krus, 2012). Lastly, emotion regulation, such as rumination, involves the strategies used by individuals to adapt the intensity and duration of emotional response (Gross, 2014).

Self‐control and response inhibition are essential for appropriate emotion expression and emotion regulation (Ochsner & Gross, 2005). Higher self‐control is associated with more effective regulation of anger (Tonnaer et al., 2016). On the other hand, lower self‐control may contribute to the development of anger rumination, which refers to continuously dwelling on angry thoughts and feelings after an anger‐provoking event (i.e., a non‐adaptive emotion regulation strategy; Lemos et al., 2021). Engaging in anger rumination further diminishes a person's self‐control capacity, creating a cycle where the likelihood of continued rumination increases (Denson, 2013). Additionally, anger rumination is associated with lower response inhibition abilities (Whitmer & Banich, 2007) and higher associations between anger rumination and inhibitory control (i.e., resistance to proactive interference) compared to associations with other cognitive control abilities (Zetsche et al., 2018). Rumination increases negative affect (Aldao et al., 2014). Anger, in this context, is a temporary reaction characterized by varying levels of emotional outbursts (Spielberg, 1999) triggered by obstacles to achieving a goal (Carver & Harmon‐Jones, 2009).

In spite of the conceptual overlap between the definitions of self‐control (typically measured through self‐report measures) and response inhibition (typically measured through cognitive tasks), previous studies revealed limited evidence of relationships between them (Allom et al., 2016; Eisenberg et al., 2019; Necka et al., 2018; Saunders et al., 2018). Therefore, rather than representing two indicators of a common higher‐order construct, self‐control and response inhibition may function as distinct entities embedded in the dynamic system of self‐regulatory control. However, it remains unclear how the mutual links between these two and other components interact along the course of emotional processes and its regulation and, in particular, in the case of anger.

The temporal network approach in psychology has the potential to advance the comprehension of both the structure and dynamics of self‐control, response inhibition, and anger (momentary states and rumination) by applying principles of complex systems and graph theory to psychological constructs, such as psychopathology, personality, and attitudes (Borsboom et al., 2021). A psychological network, also referred to as a *graph*, is composed of distinct entities of psychological variables, called *nodes*, and their connections, the *edges*. The latter express either undirected (i.e., cross‐sectional) or directed (i.e., predictive or even causal) associations between pairs of nodes. In both cases, the strength of each edge is quantified as the *partial* effect relating two nodes when the effects of all other nodes are controlled for.

Along these lines, Neubeck et al. (2022) examined the structure of cross‐sectional networks covering 12 self‐reported facets of self‐regulation (including self‐control and emotional processes as well as response inhibition). In terms of the macrostructure of the network, the authors observed stronger connections within the two clusters of self‐regulation and executive functions than between them. A psychometric conclusion that can be drawn in such a cross‐sectional configuration suggests a partial conceptual overlap of self‐regulation and executive functions (Epskamp et al., 2018). However, mechanistic conclusion for psychological networks such as the dynamics of self‐regulation and executive functions are not justified by cross‐sectional designs (Hamaker, 2012, 2023). Also, longitudinal network models have the potential to reveal the temporal dynamics between processes studied at the within‐person level (Epskamp et al., 2012). Thus, they provide a more comprehensive description of the underlying dynamic interactions between psychological variables (Borsboom et al., 2021; Bringmann et al., 2022; Fried & Cramer, 2017).

By using an intensive longitudinal approach in the analysis of naturally occurring self‐regulation, important information is gathered about its variability over time, both at the individual and group levels (Myin‐Germeys et al., 2018; Santangelo et al., 2017). Temporal networks can describe different dynamics among self‐control, response inhibition, and anger (momentary states and rumination). For example, anger rumination may predict momentary states of anger on the subsequent measurement occasions. Moreover, we can examine the automaticity within a process based by the extent to which it predicts itself over time. Specific constellations may arise when predictive relations between two processes are mediated by another process. In addition, reciprocal dynamics may occur when, for example, decreased self‐control is both a predictor and an outcome of momentary anger. Momentary anger may also interfere with youths' subsequent capacity to inhibit responses, which may then reinforce anger rumination. Taken together, such a reciprocal scenario may function as a dynamic feedback loop that can be studied using temporal network analyses. These hypothetical illustrations highlight the significance of examining the interrelationships between these processes through network analysis approach.

To the best of our knowledge, no previous study has examined the temporal dynamics of self‐control, response inhibition, and anger (momentary states and rumination) at the within‐person level. Hence, adopting a temporal within‐person approach will enable advancement of our comprehension of the nature of these processes over time (Myin‐Germeys et al., 2018). Such an approach permits the assessment of temporal sequences among theoretically linked constructs (Depp, Moore, Dev, et al., 2016; Depp, Moore, Perivoliotis, et al., 2016) within the natural context (i.e., real world) in which they occur in “real time.” Moreover, this dynamic perspective disentangles self‐reported self‐control from performance measures of response inhibition on a mechanistic level by examining not only the temporal association between them but also their distinctive role within the emotional experience of anger and of anger rumination.

From this perspective, ambulatory assessment is of particular interest and provides ecologically valid measures. Ambulatory assessment includes the experience sampling method (ESM), a method used to repeatedly assess the self‐reported thoughts, cognition, emotions, experiences, and behaviors of individual subjects in naturalistic environments on a momentary level (Myin‐Germeys & Kuppens, 2022; van Roekel et al., 2019). Thus, ambulatory assessment has the potential to capture fine‐grained intra‐individual variability over time (George et al., 2017; Myin‐Germeys et al., 2018; Myin‐Germeys & Kuppens, 2022; Russell & Gajos, 2020). Ambulatory cognitive performance (Jones et al., 2018) is also embedded in ambulatory assessment even if it is less common in previous studies. Such assessment provides valid and reliable measures of cognitive control (Moore et al., 2022).

### The current study

A few previous studies examined the interrelationships among self‐control, response inhibition, and anger (states and rumination) at the between‐person level, leaving the within‐person level and the temporal dynamics of the interrelationships largely unexplored. Thus, by applying data‐driven temporal network analysis, our aim was to identify the dynamic associations among self‐control, response inhibition, and anger experiences as they occur in real‐life settings. More specifically, we expected that self‐control and response inhibition would have distinct predictive and undirected associations with states of anger and anger rumination. In this perspective, network analyses may inform more fine‐grained theory building and mechanistic research on the antecedents of anger and self‐control, two core dimensions of behavioral problems overrepresented in boys. Therefore, this study focuses only on boys.

## MATERIAL AND METHODS

### Participants

We recruited 62 male adolescents, aged between 12 and 18 years, with a wide range of behavioral and emotional problems and who have access to a smartphone device (socio‐demographic information shown in Table 1). Potential candidates were excluded if the level of comprehension of the French language was insufficient or if they had any known diagnoses of schizophrenia, psychosis, or autism spectrum disorders at the present time or if they were under treatment with psychotropic drugs (i.e., antipsychotics), which have repercussions on behavior and emotion regulation. We advertised on dedicated websites (hospital websites and youth‐specific websites), in schools, and in residential care institutions to recruit the youths.

**TABLE 1:** Socio‐demographic characteristics (*n* = 62).

Variable	Level	*M* (SD)/% (*n*)
Age	Years	15.19 (1.53)
Socio‐economic level	Low	17.24 (10)
	Medium	37.93 (22)
	High	44.83 (26)
Nationality	Swiss	82.25 (51)
First language	French	90.23 (56)
Ongoing education	Yes	95.16 (59)

Socioeconomic status (SES) was determined based on the father's and mother's professional activities and their highest degree of education completed (Kaufman et al., 2000). Participants rated the Youth Self Report from the Child Behavior Check List (Vermeersch & Fombonne, 1997; Vreugdenhil et al., 2006) to rate externalizing symptoms (*n* = 16 with *T*‐scores ≥65) and adjustment problems (*n* = 9 with *T*‐scores ≥65) (Donado et al., 2020; Koechlin et al., 2018).

### ESM measures

*Self‐control* was assessed through four questions (*“Since the last survey … I was able to focus on the activities at hand without being distracted”*; *“I was able to follow my plans and objectives”*; *“I lost control of myself”*; *“I easily resisted the temptation/my desires of the moment”*), rated on a visual analog scale ranging from “*No, not at all*” to “*Yes, totally*” (Cronbach's *α* = .66, which is not optimal and thus should be interpreted with caution) and adapted from the revised early adolescent temperament questionnaire (Ellis & Rothbart, 2001), the Barratt Impulsiveness Scale (Rouselle & Vigneau, 2016), and the Brief Self‐Control Scale (Brevers et al., 2017).

*Response inhibition* was measured with a Go/No‐go task run on the Inquisit^©^ website. The task consisted of four blocks of 50 trials each, with 10 trials per block presenting no‐go stimuli (20% of all trials). These blocks were preceded by a training block of 10 trials. The participants performed the task on their own smartphones. Each trial began with a fixation cross (“+”), followed by the stimuli (either go or no‐go stimuli), which appeared in any one of six positions on the screen (for 1200 ms). The participants received feedback after each trial (“correct” or “incorrect”). The participants were instructed to respond as quickly and accurately as possible. Our primary outcome was accuracy in the no‐go trials (no response to no‐go stimulus). The Spearman–Brown coefficient (between odd and even days) was 0.96, indicating excellent reliability.

We assessed momentary states of anger by asking the participants how angry (“*Now, I feel: angry/frustrated*”) they felt, rated on a visual analog scale ranging from “*No, not at all*” to “*Yes, totally.*” Anger rumination (one nonadaptive emotion regulation strategy) was measured with two items (*“Since the last survey … I ruminated/thought about my past angry experiences”; “I analyzed the events that made me angry”*), adapted from the anger rumination scale (Sukhodolsky et al., 2001) rated on the same visual analog scale (Cronbach's *α* = .79).

### Procedure

The study was authorized by the ethics committee of the canton of Vaud (#2019‐02318). Each adolescent and his legal representative signed a written consent form to participate. The ESM protocol consisted of two measures per day through nine consecutive weekdays (we excluded the weekend to assure homogenous sampling regarding the different variations of daily activities between weekends and weekdays). SMS messages prompted the participants to respond to the link to the Redcap^®^ (Harris et al., 2009, 2019) survey (for the self‐report section). Other SMS messages gave the link to the Inquisit^©^ (Millisecond Software, MA) website to perform the Go/No‐go task (see below for details) on their own smartphones. The SMS messages were sent at 7:00 a.m. and 4:00 p.m. to cover the day. The completion of each assessment lasted about 10 min (4 min for the self‐report part and 6 min to perform the Go/No‐go task). The participants received monetary incentives for their participation. We observed a mean compliance rate of 85% for the self‐report measures and 79.3% for the Go/No‐go task, which represents high compliance (for reviews, see Jones et al., 2019; van Roekel et al., 2019; Wen et al., 2017; Wrzus & Neubauer, 2022).

### Data analysis

Descriptive statistics characterized the baseline measures of the sample. The mean and standard deviation (SD) were reported for the continuous variables, whereas the number of observations and percentages were used for the categorical variables. Descriptive statistics were also provided for self‐control, response inhibition, momentary state of anger, and anger rumination that refer to the four nodes of the network in the analyses (on the overall sample and separately for the morning and afternoon measures).

With the intensive longitudinal data from the ambulatory assessment, dynamic network analysis was undertaken to study the interrelationships among the four processes at the within‐ and between‐person levels (Borsboom et al., 2021; Bringmann et al., 2013, 2022). With time series data, we can estimate the temporal network via fitting vector of auto‐regression (VAR) models. However, our data structure necessitates the use of multilevel VAR models that extends the traditional VAR model to account for hierarchical or nested structures in the data. This method allows for investigating the dynamics among the nodes within and across different individuals, providing insights into the complex relationships in multilevel systems. Prior to fitting the models, the temporal dependence of order 1 is considered as the multilevel VAR models only allowed to examine the effect of the measurement in the morning on those in the afternoon. This was necessary to avoid measurement bias from the night before that can have a different impact on the next measurement in the morning (Bringmann et al., 2022).

In this context, a multilevel model with autoregressive components is fitted separately for each node (one of the four processes) controlling for the value of all the other nodes (all the other processes) from the previous time point as well as the person‐wise mean of these variables. This resulted in the estimation of two networks: (a) the within‐person temporal network (i.e., presenting the effect of the measurement in the morning predicting the measurements in the afternoon); and (b) the between‐person network that expressed the covariation of the constructs on average in the data. The remaining residuals of these models were then used in the second step to estimate the within‐person concurrent effect by fitting a multilevel model at each node. This was done to first extract the temporal and between‐person effect from the data and then to correctly specify the within‐person effect at one time point.

For within temporal person models, the result of the estimated fixed effects, SDs, and the *p*‐values are reported in Table S1. The results of the concurrent within‐ and between‐person networks were reported as partial correlation coefficients and their *p*‐value in Tables S2 and S3, respectively.

**FIGURE 1:**
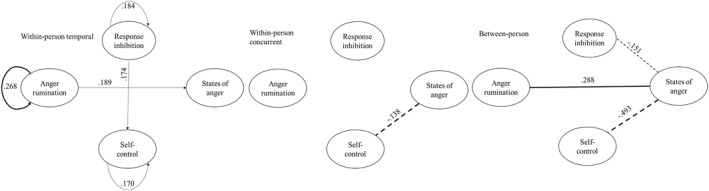
Network analyses. Temporal within‐person network examining the effect of the measure of the morning on those in the afternoon (lagged model; arrows representing the direction of the link, from morning to afternoon assessment). The concurrent (cross‐sectional) within‐person network modeled the link between the measures at a time‐point at the within‐person level. The between‐person network model refers to the interrelationships of the variable according the between‐person variability. Plain arrows/lines refer to positive association whereas dashed lines refer to negative associations. The width of the lines or arrows represents the strength of the association.

In plotting the networks (Figure 1), only edges that surpass the significance threshold were shown (for which the adjusted *p*‐value < .05). For the contemporaneous and between‐person network, the rule of “or” is used to decide on depicting the significant partial correlations. That is to say if, in one way of the fitted model, the partial correlation coefficient is significant, then the edge is considered significant. All the *p*‐values were adjusted using the false discovery rate to account for multiple testing. The network analyses were computed in the R environment for statistical computing (version 4.1.0) (Team R Core, 2018) using the package “mlVAR.”

## RESULTS

### Descriptive

Table 2 reports the descriptive statistics for each variable of interest for all measures as well as separately for the morning and afternoon assessments. No difference was observed between the morning and afternoon measures.

**TABLE 2:** Descriptive.

	All measures		Morning		Afternoon		*p* ^a^
	*M*	SD	*M*	SD	*M*	SD	
Self‐control	74.61	21.35	74.67	20.96	74.55	21.75	*ns*
Response inhibition	56.14	23.80	57.23	24.01	54.97	23.54	*ns*
States of anger	10.97	21.95	10.36	21.21	11.57	22.67	*ns*
Anger rumination	12.59	23.01	13.54	23.68	11.64	22.30	*ns*

*Note*: Self‐control: mean of four items, possible range from 0 to 100. Response inhibition, data expressed as percentage, possible range from 0 to 100. States of anger: subjective score from visual analog scale, possible range from 0 to 100. Anger rumination: mean of two items, possible range 0–100.

^a^ Comparison of morning and afternoon measure.

### Within‐person temporal network

The network analysis assessing within‐person temporal associations revealed that for an adolescent, response inhibition (*β* = .184, SD = .240, *p* = .005), anger rumination (*β* = .268, SD = .415, *p* = .002), and self‐control (*β* = .170, SD = .065, *p* = .028) were auto‐correlated (i.e., morning measures are associated with afternoon measures). The adolescent's states of anger in the morning were not related to their states of anger in the afternoon (*β* = −.006, SD = .089, *p* = .939). When the adolescent reported more anger rumination in the morning, they reported higher states of anger in the afternoon (*β* = .189, SD = .302, *p* = .028). States of anger in the morning were not associated with any other processes in the afternoon. Adolescents performing well in response inhibition in the morning reported higher self‐control in the afternoon (*β* = .174, SD = .141, *p* = .025) but not the opposite (see Figure 1).

#### Within‐person concurrent network

The second network explored cross‐sectional associations within the same time point measure for an adolescent (i.e., within‐person level). This analysis revealed that, on average, an adolescent reporting higher states of anger reported lower self‐control (partial‐*r* = −.138, *p* = .022).

### Between‐person network

At the between‐person level, adolescents who reported lower states of anger performed better in response inhibition on average (partial‐*r* = −.150, *p* = .027) and reported higher self‐control (partial‐*r* = −.491, *p* = .005). By contrast, adolescents who reported higher states of anger also reported higher anger rumination (partial‐*r* = .291, *p* = .029).

## DISCUSSION

This study aimed to examine the temporal dynamics among self‐control, response inhibition, and anger (i.e., momentary states and rumination) using an experience sampling design in male adolescents with a wide range of behavioral difficulties. The results revealed the following at the within‐person level: (a) stability across the day for self‐control, response inhibition, and anger rumination; (b) higher response inhibition in the morning related to higher self‐control in the afternoon and more anger rumination in the morning leading to higher momentary states of anger in the afternoon; and (c) in a given moment (i.e., cross‐sectional associations), higher states of anger related to lower self‐control. Moreover, at the between‐person level, we observed that higher states of anger were related to lower response inhibition and self‐control but to higher anger rumination.

We observed that anger rumination (i.e., automatically rethinking events eliciting anger or failure of emotion regulation) led, at a later time point, to higher states of anger, indicating that anger rumination precedes anger. This result is in line with the proposed role of rumination in understanding psychopathology (Ehring, 2021), such as depression, anxiety, post‐traumatic stress disorder, eating disorders, or substance use disorders (Ehring & Watkins, 2008; Nolen‐Hoeksema et al., 2008). Our results are in line with those of previous work proposing that rumination may increase negative affect (Aldao et al., 2014) through metacognitive beliefs (Wells, 2010). Notice that negative affect such as anger (or irritability), when not appropriately regulated, represent one of the main risk factors for developing (Habersaat et al., 2018; Urben et al., 2017) and maintaining (Urben et al., 2022) behavioral problems. This result sheds some light on an important precursor that may present an important target of intervention.

In line with previous studies (Allom et al., 2016; Eisenberg et al., 2019; Necka et al., 2018; Saunders et al., 2018), we noticed no cross‐sectional (in a given moment both at the within‐ and between‐person levels) relationships between response inhibition measured through an ambulatory cognitive task and self‐reported self‐control both at the within‐ and between‐person levels. We found that the lack of association observed from both the within‐ and between‐person levels also apply to the concurrent residual associations. Some distinctions might help understand this result (Wennerhold & Friese, 2020). The self‐report measure of self‐control taps the average representation of the ability to control oneself for some hours, whereas the performances in the Go/No‐go task assess cognitive control “at moment” skills in real time (Jones et al., 2013). The self‐reported measure of self‐control represents a trait‐like characteristics and performance in the Go/No‐go task reflects cognitive control at one point in time. Self‐control encompasses motivational aspects (or emotional ones) and future planning (i.e., the need to stick to goals or plans), whereas the Go/No‐go task measures purer cognitive (or executive) processes.

In contrast, when we assessed the temporal dynamics between self‐control and response inhibition, we noticed that higher performances in cognitive control in the morning lead to higher self‐reported self‐control in the afternoon. Taking an integrative perspective of self‐regulation (Bailey & Jones, 2019), we might hypothesize that higher‐order processes (i.e., self‐control) are sustained by core processes (i.e., response inhibition). According to this integrated model of self‐regulatory control processes, the domains of regulation as well as the level of processes are hierarchically organized. Executive functions (e.g., response inhibition) refer to core processes across domains supporting higher‐order regulatory processes (i.e., self‐control) (Bailey & Jones, 2019). During development, the regulatory domains that emerge are gradually elaborated, coordinated, and consolidated in the behavioral repertoire. Regulatory skills are gradually articulated and integrated with domain‐specific learning to produce increasingly complex behavior and finally solidified in “Regulatory Gestalt” (Bailey & Jones, 2019). This is in line with a previous study reporting closer relationships within the self‐regulatory control processes network with age (Neubeck et al., 2022). Our results provide, thus, a clearer understanding of the temporal interrelationships between these two components of self‐regulatory control processes in the cognitive domain.

Finally, at the between‐person level, we observed that higher anger is associated with lower self‐control and response inhibition. To understand these results, we could relate to the dual competition model (Pessoa, 2009), which posits that emotion and cognition share a common pool of resources. Thus, when emotions use these resources (by higher anger), fewer resources are available for other effortful processes (i.e., response inhibition and self‐control).

Our results are in line with those of a study examining networks among self‐control, emotion regulation, and executive function through development (Neubeck et al., 2022). However, we not only specify the interrelationships cross‐sectionally at the between‐person level but also extend it to the temporal dynamics at the within‐person level. Consistent with our results, Neubeck et al. (2022) reported more relationships between self‐control and emotion regulation compared to the associations with executive function (including response inhibition). This study had, however, been conducted on cross‐sectional data at the between‐person level. Thus, our study goes a step further by examining this association at the within‐person level and the temporal dynamics of these associations.

Our results may contribute to developing ecological momentary interventions (EMI), which refer to treatments provided to youths in their ecological environment and in their everyday lives (Heron & Smyth, 2010). These interventions may be presented as mobile applications that records the patient's behaviors, cognition, and emotions and that are able to give, at a specific time, the appropriate support or help. In this line, just‐in‐time adaptive interventions refer to EMI coupled with ambulatory assessments. This allows supplying a personalized intervention based on real‐time assessment (Patrick et al., 2005) providing the appropriate amount of support at the right time (Nahum‐Shani et al., 2017). Such innovative interventions may increase the access to treatment (Shiffman et al., 2008) by providing the most appropriate intervention at the best time (for a scoping review, see Balaskas et al., 2021). For instance, it seems that detecting higher anger rumination and lower performance in response inhibition to intervene just in time in these processes may reduce their negative impact on later higher anger and lower self‐control, respectively.

Some limitations of the present study should be taken into account. This study was encompassed in a pilot project examining the role of self‐regulatory processes in externalizing symptoms. We included only male adolescents (to have a more homogenous sample). Therefore, these findings have to be replicated with larger samples including female adolescents. Indeed, the interrelationships may be different in female participants, especially since anger and anger rumination may play a different role in women compared to men. Moreover, we focused on four specific components of self‐regulation. Therefore, further studies should include more processes, such as other emotion regulation strategies, impulsivity, and autonomic regulation (an important biomarker in the context of behavioral difficulties; Beauchaine & Thayer, 2015). Moreover, this study reported important interrelationships among mechanisms at the micro‐level. Although these mechanisms may be linked to the development of externalizing symptoms, future studies should examine if these mechanisms are related to changes over time in externalizing symptoms.

## CONCLUSION

This study applied an innovative approach—namely, temporal network analysis—to understand the temporal dynamics of the associations among self‐control, response inhibition, and anger at both the within‐ and between‐person levels. The study provides important evidence to outline the temporal relationships and the integration of the within‐ and between‐person levels.

## AUTHOR CONTRIBUTIONS

**Fiorella Turri**: Conceptualization; visualization; writing – original draft; writing – review & editing. **Andrew jones**: Conceptualization; methodology; resources; software; writing – review & editing. **Lauriane Constanty**: Data curation; investigation; writing – review & editing. **Setareh Ranjbar**: Conceptualization; data curation; formal analysis; methodology; software; writing – review & editing. **Konstantin Drexl**: Conceptualization; writing – review & editing. **Giorgia Miano**: Investigation; writing – review & editing. **Caroline Lepage**: Conceptualization; methodology; writing – review & editing. **Kerstin Jessica Plessen**: Conceptualization; methodology; project administration; resources; supervision; validation; writing – review & editing. **Sébastien Urben**: Conceptualization; data curation; funding acquisition; investigation; methodology; project administration; resources; supervision; writing – original draft; writing – review & editing.

## CONFLICT OF INTEREST STATEMENT

The authors have declared that they have no competing or potential conflicts of interest.

## ETHICAL CONSIDERATIONS

The study was authorized by the ethics committee of the canton of Vaud (#2019‐02318).

## Supporting information

Supporting Information S1

## Data Availability

The original contributions presented in the study are included in the article further inquiries can be directed to the corresponding author.
