# Supplementary material for: Self‐regulatory control processes in youths: A temporal network analysis approach
Source: JCPP Adv. 2023 Sep 30;4(1):e12200. doi: 10.1002/jcv2.12200 (PMC10933645; doi:10.1002/jcv2.12200)
Supplement: Supplementary file 1 — Supporting Information S1 [file JCV2-4-e12200-s001.docx]

**Supporting Information**

**Table S1.** Detailed results of the within-person temporal network

| **Morning** | **Afternoon** | ***β*** | **SD** | **P-value** |
| --- | --- | --- | --- | --- |
| Response inhibition | Response inhibition | 0.184 | 0.240 | .005 |
| Response inhibition | Anger rumination | -0.035 | 0.341 | .668 |
| Response inhibition | Self-control | 0.174 | 0.141 | .025 |
| Response inhibition | States of anger | -0.025 | 0.01 | .770 |
| Anger rumination | Response inhibition | 0.028 | 0.089 | .514 |
| Anger rumination | Anger rumination | 0.268 | 0.415 | .002 |
| Anger rumination | Self-control | 0.081 | 0.014 | .148 |
| Anger rumination | States of anger | 0.189 | 0.302 | .028 |
| Self-control | Response inhibition | 0.029 | 0.120 | .617 |
| Self-control | Anger rumination | -0.026 | 0.149 | .708 |
| Self-control | Self-control | 0.17 | 0.065 | .028 |
| Self-control | States of anger | 0.137 | 0.245 | .165 |
| States of anger | Response inhibition | 0.086 | 0.033 | .063 |
| States of anger | Anger rumination | -0.017 | 0.238 | .806 |
| States of anger | Self-control | -0.084 | 0.050 | .196 |
| States of anger | States of anger | -0.006 | 0.089 | .939 |

Note : SD : Standard Deviation.

**Table S2.** Detailed results of the within-person concurrent (cross-sectional) network

| **Association** | **partial-*r*** | **P-value** |
| --- | --- | --- |
| Anger rumination - Response inhibition | 0.043 | .358 |
| Self-control - Response inhibition | 0.091 | .095 |
| Self-control - Anger rumination | 0.030 | .488 |
| States of anger - Response inhibition | 0.003 | .931 |
| States of anger - Anger rumination | 0.130 | .099 |
| States of anger - Self-control | -0.138 | .022 |

Note. Partial-*r*: partial correlation coefficients

**Table S3.** Detailed results of the between-person network

| **Association** | **partial-*r*** | **P-value** |
| --- | --- | --- |
| Anger rumination - Response inhibition | 0.058 | .412 |
| Self-control - Response inhibition | -0.078 | .441 |
| Self-control - Anger rumination | -0.221 | .102 |
| States of anger - Response inhibition | -0.151 | .027 |
| States of anger - Anger rumination | 0.288 | .029 |
| States of anger- Self-control | -0.493 | <.001 |

Note. Partial-*r*: partial correlation coefficients
